# Multiomics-based characterization of specialized metabolites biosynthesis in *Cornus Officinalis*

**DOI:** 10.1093/dnares/dsaa009

**Published:** 2020-05-19

**Authors:** Amit Rai, Megha Rai, Hidetaka Kamochi, Tetsuya Mori, Ryo Nakabayashi, Michimi Nakamura, Hideyuki Suzuki, Kazuki Saito, Mami Yamazaki

**Affiliations:** 1 Graduate School of Pharmaceutical Sciences, Chiba University, Chiba 260-8675, Japan; 2 Plant Molecular Science Center, Chiba University, Chiba 260-8675, Japan; 3 RIKEN Center for Sustainable Resource Science, 1-7-22 Suehiro-cho, Tsurumi-ku, Yokohama 230-0045, Japan; 4 Department of Research and Development, Kazusa DNA Research Institute, Kisarazu, Chiba 292-0818, Japan

**Keywords:** Cornus officinalis, specialized metabolites, iridoids, gallotannins, integrative omics

## Abstract

*Cornus officinalis*, an important traditional medicinal plant, is used as major constituents of tonics, analgesics, and diuretics. While several studies have focused on its characteristic bioactive compounds, little is known on their biosynthesis. In this study, we performed LC-QTOF-MS-based metabolome and RNA-seq-based transcriptome profiling for seven tissues of *C. officinalis*. Untargeted metabolome analysis assigned chemical identities to 1,215 metabolites and showed tissue-specific accumulation for specialized metabolites with medicinal properties. *De novo* transcriptome assembly established for *C. officinalis* showed 96% of transcriptome completeness. Co-expression analysis identified candidate genes involved in the biosynthesis of iridoids, triterpenoids, and gallotannins, the major group of bioactive metabolites identified in *C. officinalis*. Integrative omics analysis identified 45 cytochrome P450s genes correlated with iridoids accumulation in *C. officinalis.* Network-based integration of genes assigned to iridoids biosynthesis pathways with these candidate CYPs further identified seven promising CYPs associated with iridoids’ metabolism. This study provides a valuable resource for further investigation of specialized metabolites’ biosynthesis in *C. officinalis.*

## 1. Introduction

*Cornus officinalis*, a deciduous tree from *Cornaceae* family and indigenous to eastern Asia, is well known for its medicinal properties.^1^ Fruits of *C. officinalis,* Corni Fructus, also referred as ‘Sanshuyu’ in Japanese and ‘shān zhū yú’ in Chinese, is an essential ingredient of several Kampo and Traditional Chinese medicinal formulations including Hachimi-gan and Rokumi-gan, among others.^2^ It is characterized to nourish liver and kidney and is often used for the treatment of kidney diseases, including diabetic nephropathy. Pharmacological studies using metabolite extracts from its fruits have shown to possess a wide range of therapeutic properties, including anti-inflammatory, anti-oxidants, anti-bacterial, anti-dementia, anti-hyperglycaemia, anti-aging, and neural protection.^3^^,^^4^ While pericarp of *C. officinalis* is primarily used as ingredients for different medicinal preparations, several studies have also reported biological activities for the leaf and seed extracts.^5–7^ Because of the wide range of medicinal properties, *Corni Fructus* is one of the 25 plant-based drugs most commonly used in China, Japan, and Korea.^8^

Till date, 305 metabolites from broad chemical families, including alkaloids, iridoids, flavones, terpenoids, polysaccharides, tannins, and essential oils, have been reported from different tissues of *C. officinalis*.^3^ Among these specialized metabolites, iridoids are regarded as the principal constituents of its medicinal properties.^4^ The Chinese pharmacopeia considers iridoids, namely, morroniside and loganin, to constitute a minimum of 1.2% of the extracted metabolites from the *C. officinalis* tissues for being regarded as suitable for use in pharmaceutical applications.^3^ Iridoid glycosides from *C. officinalis* extracts such as loganin, morroniside, and cornuside are known to possess neuroprotective, cardioprotective, hypoglycaemic, anti-osteoporotic, anti-oxidant, anti-tumour, anti-diabetic, and anti-dementia activities.^9–12^ Triterpenoids, including ursolic acid and oleanolic acid, have been reported to possess anti-cancer, anti-inflammatory, anti-oxidative, hepatoprotective, anti-ulcer, hypolipidemic, and anti-atherosclerotic activity.^13–15^ Tannins, including tri-*O-*galloyl-β-d-glucose and tetra-*O-*galloyl-β-d-glucose, are reported to have neuroprotective activity, while isoterchebin, tellimagrandin I, and gallic acid are known to prevent Alzheimer’s disease.^16–18^

Despite being a rich source of diverse metabolic content of known pharmacological properties, limited omics-resources for *C. officinalis* have restricted applications of biotechnology tools for species improvement and elucidation of biosynthetic pathways of these valuable metabolites. RNA-seq-based *de novo* transcriptome assemblies of non-model medicinal plants have proven to be a valuable genomic resource to facilitate the identification of key enzymes involved in the biosynthesis of target metabolites.^19^^,^^20^ RNA-seq approach assigns transcripts to different enzyme classes and families based on sequence similarity. This approach leads to multiple transcripts being assigned to a single biochemical reaction step^21^^,^^22^ due to the genomic redundancies in the plant kingdom. Hence, the homology-based approach by itself is not enough for narrowing down or prioritizing candidate genes involved in the biochemical reactions for further functional characterization.

**Figure 1:**
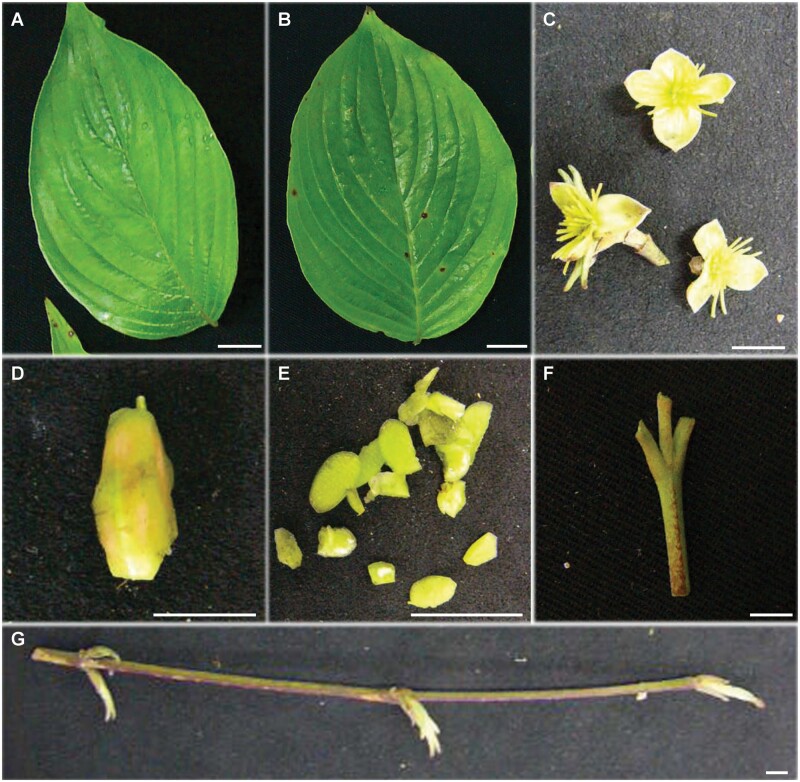
Tissues used for the systems-level study on the biosynthesis of specialized metabolites in *Cornus officinalis*. (A) young leaf; (B) mature leaf; (C) inflorescence; (D) seed; (E) pericarp; (F) young stem; and (G) mature stem; the bars represent 1 cm.

Co-expression analysis as a mean to prioritize genes for functional characterization has served plant biologist well and have contributed to our present understanding of specialized metabolite biosynthesis pathways such as iridoids and triterpenoids among others.^19^^,^^23^ Biosynthesis of specialized metabolites involves a minimal set of core molecules, which then serve as the substrate for enzyme families such as cytochrome P450s (CYPs), glycosyltransferases, and acyltransferases among others to result in the enormous chemodiversity, a characteristic of plant metabolome.^19^^,^^24^ Integrating co-expression with other omics datasets further narrow down candidate gene lists by excluding false-positive gene candidates for functional characterization. Co-expression analysis, complemented with correlation-based integration of metabolome and transcriptome dataset, is established as an efficient approach facilitating the discovery of novel genes.^25–27^ While several studies have adopted targeted analysis to identify metabolites present in *C. officinalis,*^3^^,^^4^ information on the differential accumulation of specialized metabolites and biosynthesis across different tissues continues to be limited*.* Here, we performed an untargeted metabolome analysis of *C. officinalis* using seven tissues, namely, young leaf, mature leaf, young stem, mature stem, pericarp, seed, and inflorescence (Fig. 1). The same set of seven tissues was used to derive *de novo* transcriptome assembly of *C. officinalis*, and subsequently for the co-expression analysis to characterize the biosynthetic pathways of its therapeutic metabolites. Using untargeted metabolome analysis, we explored the differential distribution of specialized metabolite classes, including iridoids, triterpenoids, and gallotannins, in *C. officinalis.* Further, sequence-similarity-based annotation of the transcriptome dataset together with their co-expression analysis was used to assign candidate genes to their biosynthetic pathway. Homology-based sequence analysis coupled with transcriptome and metabolome data integration allowed us to propose candidate cytochrome P450s associated with iridoids metabolism. Connection network-based correlation analysis between iridoids and cytochrome P450s together with the assigned biosynthetic genes identified seven CYPs as the strongest candidate among the identified CYPs for further functional characterization. Our study thus provided valuable insights into the specialized metabolites biosynthetic pathway in *C. officinalis.* The metabolome and transcriptome resources established in this study, together with proposed candidate CYPs will facilitate better understanding of the biosynthesis and regulation of metabolic pathways present in *C. officinalis.*

## 2. Materials and methods

### 2.1. Plant material

*Cornus officinalis* plants were maintained under natural conditions at the medicinal garden, Chiba University. All the seven tissues of *C. officinalis,* namely, young leaf, mature leaf, young stem, mature stem, pericarp, seed, and inflorescence (Fig. 1) were harvested on ice and immediately snap-frozen using liquid nitrogen. The harvested tissues were then stored at −80°C until further processing.

### 2.2. Untargeted metabolite profiling

The tissues were freeze-dried using a freeze dryer (FDU-2200, Tokyo Rika-Kikai, Japan). The samples were added with 50 μl of HPLC-grade methanol (Wako, Japan) and 2.5 μM of lidocaine (Wako, Japan) per milligram of dry weight. Further, the mixture was homogenized using mixer mill (MM300, Retsch) with zirconia beads for 7 min at 18 Hz, 4°C, and then centrifuged at 15,000 ***g*** for 10 min. The supernatant was subsequently filtered using a 96-well Oasis HLB μElution plate (Waters Inc., USA). The MS and MS/MS (MS^2^) datasets were acquired as described previously.^28^ The extracts (1 μl) were analysed using LC-QTOF-MS (LC, Waters Acquity UPLC system; MS, Waters Xevo G2 Q-Tof). Analytical conditions were as follows; LC: column, Acquity bridged ethyl hybrid C18 (1.7 μm, 2.1 mm × 100 mm, Waters); solvent system, solvent A (water including 0.1% formic acid) and solvent B (acetonitrile including 0.1% formic acid); gradient programme, 99.5%A/0.5%B at 0 min, 99.5%A/0.5%B at 0.1 min, 20%A/80%B at 10 min, 0.5%A/99.5%B at 10.1 min, 0.5%A/99.5%B at 12.0 min, 99.5%A/0.5%B at 12.1 min and 99.5%A/0.5%B at 15.0 min; flow rate, 0.3 ml/min at 0 min, 0.3 ml/min at 10 min, 0.4 ml/min at 10.1 min, 0.4 ml/min at 14.4 min, and 0.3 ml/min at 14.5 min; column temperature, 40°C; MS detection: capillary voltage, +3.0 keV, cone voltage, 25.0 V, source temperature, 120°C, desolvation temperature, 450°C, cone gas flow, 50 l/h; desolvation gas flow, 800 l/h; collision energy, 6 V; mass range, *m/z* 100‒1,500; scan duration, 0.1 s; inter-scan delay, 0.014 s; data acquisition, centroid mode; polarity, positive: scan duration, 1.0 s; inter-scan delay, 0.1 s. MS2 data were acquired in the ramp mode as the following analytical conditions: (1) MS: mass range, *m/z* 50–1,500; scan duration, 0.1 s; inter-scan delay, 0.014 s; data acquisition, centroid mode; polarity, positive/negative; and (2) MS^2^: mass range, *m/z* 50–1,500; scan duration, 0.02 s; inter-scan delay, 0.014 s; data acquisition, centroid mode; polarity, positive/negative collision energy, ramped from 10 to 50 V. The MS^2^ spectra of the top 10 ions, with counts over 1,000 in MS scan, were acquired and moved further to the next top 10 ions based on MS-scan at a given run-time, while the acquisition was not performed if the ion intensity was <1,000. Data were processed using MS-DIAL^29^ with default parameters. Metabolite peaks were normalized through comparison with lidocaine (internal standard) and were used for Principal Component Analysis (PCA). To assign putative chemical identities to the acquired metabolite peaks, they were mapped onto the KNApSAcK database^30^ and Kyoto Encyclopedia of Genes and Genomes (KEGG) pathway database.^31^

For MS^2^-based validation of metabolites’ identity, we first prepared a comprehensive list of metabolites being previously reported in the *Cornus* genus and used it as a database to explore mass-features identified in *C. officinalis*. Subsequently, mass-features with mass-error of 10 ppm or less, when compared with previously reported metabolites, were selected and analysed further by MS-FINDER.^32^ Chemical identities were assigned to the mass-features by matching the MS^2^ fragmentation pattern with the MS^2^ ions of the corresponding metabolite as reported in previous studies or as predicted by *in silico* fragmentation by MS-FINDER as described elsewhere.^33^

### 2.3. RNA isolation, cDNA synthesis, and library construction

The tissue samples were homogenized for RNA extraction. RNA samples with RNA integrity number over 8.0 were used for cDNA library synthesis. The extraction of RNA, RNA integrity analysis to check the quality of RNA, and synthesis of the corresponding cDNA libraries were performed as described elsewhere.^34^

### 2.4. Illumina sequencing and transcriptome assembly

The cDNA libraries generated for different tissues of *C. officinalis* were sequenced using Illumina HiSeq™ 2000 (Illumina Inc., USA) to obtain paired-end read with an average read-length of 101 bps. The construction of cDNA libraries and their sequencing were performed at Kazusa DNA Research Institute, Japan. The raw sequence reads were then pre-processed to remove adaptor sequences, low-quality reads, short-read as well as ambiguous read sequences, using Trimmomatic programme.^35^ The high-quality reads were subsequently used to generate *de novo* transcriptome assembly.

For the generation of *de novo* transcriptome assembly, three popular assemblers, namely, CLC genomics workbench (https://www.qiagenbioinformatics.com/, 8 May 2020, date last accessed), Trinity^36^ with default parameters, and SOAPdenovo-Trans^37^ with six different k-mer sizes, namely, 31, 41, 51, 63, 71, and 91, were used. The transcriptome assembly resulting from SOAPdenovo-Trans with k-mer 41 showed best assembly statistics compared with other used k-mers and was selected for further use. The SOAPdenovo-Trans assembly (k-mer size 41) was concatenated with individual assembly derived from Trinity and CLC genomics workbench (Table 1)*.* The concatenated transcriptome assembly was further processed using CD-HIT-EST^38^ to remove any sequence redundancy, as described previously.^34^ For assessing the completeness of *C. officinalis* transcriptome assembly, we performed Benchmarking Universal Single-Copy Orthologs (BUSCO v.3.0.2) analysis^39^^,^^40^ using embryophyta_odb10 database.

**Table 1:** Summary of assembly statistics for *de novo* transcriptome assembly of *Cornus officinalis* based on three popular assemblers and their combination

	Kmer	No. of contigs	N50	Average length	Median length	Max length	*n*>500	*n*>1,000	Total (bp)
CLC	20	146,234	1,126	719	408	15,296	58,038 (39.7%)	28,711 (19.6%)	105,178,010
Trinity	25	404,394	1,230	734	403	15,776	165,229 (40.9%)	86,837 (21.5%)	296,953,277
SOAP*de novo*-*trans*	31	147,639	1,239	720	384	15,241	59,300 (40.2%)	31,550 (21.4%)	106,284,821
	41	148,423	1,257	715	373	15,334	57,918 (39.0%)	31,474 (21.2%)	106,155,312
	51	146,829	1,212	687	355	15,635	54,376 (37.0%)	29,330 (20.0%)	100,898,210
	63	135,683	1,171	661	334	15,335	47,621 (35.1%)	25,858 (19.1%)	89,665,413
	71	109,186	1,214	695	353	15,321	41,108 (37.6%)	22,627 (17.8%)	75,905,071
	91	28,652	1,415	936	650	12,222	16,748 (58.5%)	9,866 (34.4%)	26,826,153
CLC_Trinity_SOAP*de novo* (kmer41)_CD-HIT-EST	N.A.	304,371	1,250	742	404	15,776	124,460 (40.9%)	65,774 (21.6%)	22,575,399

### 2.5. Functional annotation, classification of assembled unigenes, and KEGG pathway mapping

For annotation of the *de novo* transcriptome assembly of *C. officinalis,* Blastx-based homology search was performed against the NCBI-non-redundant (NCBI-nr) database with an E-value cut-off as 1E-5, and the maximum number of hits set as 20. The top-blast hit was used to annotate the *de novo* transcriptome assembly. Subsequently, the associated EC number, GO terms, and KEGG pathway-based annotation was retrieved using BLAST2GO software.^41^

### 2.6. Microsatellite detection

The *de novo* transcriptome assembly of *C. officinalis* was analysed to identify the composition, frequency, and distribution of simple sequence repeats (SSRs) using MISA software.^42^ The search parameters were similar to as described elsewhere.^34^ The identified microsatellites and identification summary are provided in Table 2 and Supplementary Table S5.

**Table 2:** Statistics of simple sequence repeats (SSRs) detected in *Cornus officinalis*

Summary of identified SSRs in the *Cornus officinalis de novo* transcriptome assembly	
Total number of sequences examined	304,371
Total size of examined sequences (bp)	225,753,992
Total number of identified SSRs	95,533
Number of SSR containing sequences	70,222
Number of sequences containing >1 SSR	18,601
Number of SSRs present in compound formation	10,953
Distribution of different repeat type classes identified in the *Cornus officinalis de novo* transcriptome assembly	
Mono-nucleotides	38,599
Di-nucleotides	45,827
Tri-nucleotides	9,802
Tetra-nucleotides	712
Penta-nucleotides	168
Hexa-nucleotides	425

### 2.7. Expression analysis and assigning unigenes to specialized metabolite biosynthesis pathway

The processed RNA-seq reads for an individual tissue were mapped to the *de novo* transcriptome assembly of *C. officinalis* using Bowtie 2.0 programme.^43^ Subsequently, RSEM programme^44^ was used to estimate transcripts expression as Fragments Per Kilobase of transcript per Million mapped reads (FPKM) values, which were subsequently used to calculate the correlation between transcriptome profiling dataset of all seven tissues. Candidate genes for iridoids, triterpenoids, and gallotannins biosynthesis pathways were assigned using sequence homology and co-expression analysis, as previously described.^45^ Briefly, unigenes annotated as enzymes based on sequence similarity for a biosynthesis pathway under consideration were selected and used for co-expression analysis. Hierarchical clustering using the co-expression matrix identified a single gene cluster representing all known enzymes for an individual biosynthesis pathway. Previous studies have shown the co-expression of genes associated with iriroids,^46–49^ triterpenoids,^34^^,^^50–52^ and several others specialized metabolites biosynthetic pathways. Therefore, unigenes from these highly co-expressed gene clusters were assigned to the respective biosynthesis pathways.

### 2.8. Integrative data analysis

The transcripts annotated as cytochrome P450 with a length of over 500 bp and expression in FPKM values over five in at least one of the seven tissues were filtered. Subsequently, the Spearman matrix-based correlation analysis was performed between the filtered CYPs and identified metabolites classified as iridoids in this study. For this, the expression data of selected CYPs across seven tissues were used together with the intensities for iridoids (as total peak area) to calculate the Spearman correlation coefficient. The CYPs with a higher correlation coefficient (*R*^2^>0.85) was selected as the candidate CYPs. Further, the correlation of those candidate CYPs was calculated with the genes assigned to the iridoid biosynthetic pathway. Cytoscape^53^ (version 3.7.2) was used to visualize the relationship between the iridoids and candidate CYPs.

### 2.9. Phylogenetic analysis

The selected candidate CYPs were translated to their corresponding protein using the Translate tool from ExPASy (https://web.expasy.org/translate/, 8 May 2020, date last accessed) by selecting the translation frame that resulted in the longest amino acid sequence while starting with methionine. Protein sequences of the candidate transcripts together with representatives of major CYP families reported in plants were aligned using MUSCLE programme,^54^ and evolutionary history was inferred using the Maximum Likelihood method adopting Dayhoff *w*/freq model using MEGA X software,^55^ with bootstrap values of 1,000.

## 3. Results and discussion

### 3.1. Untargeted metabolic profiling revealed complex metaboconstituents of *Cornus officinalis*

**Figure 2:**
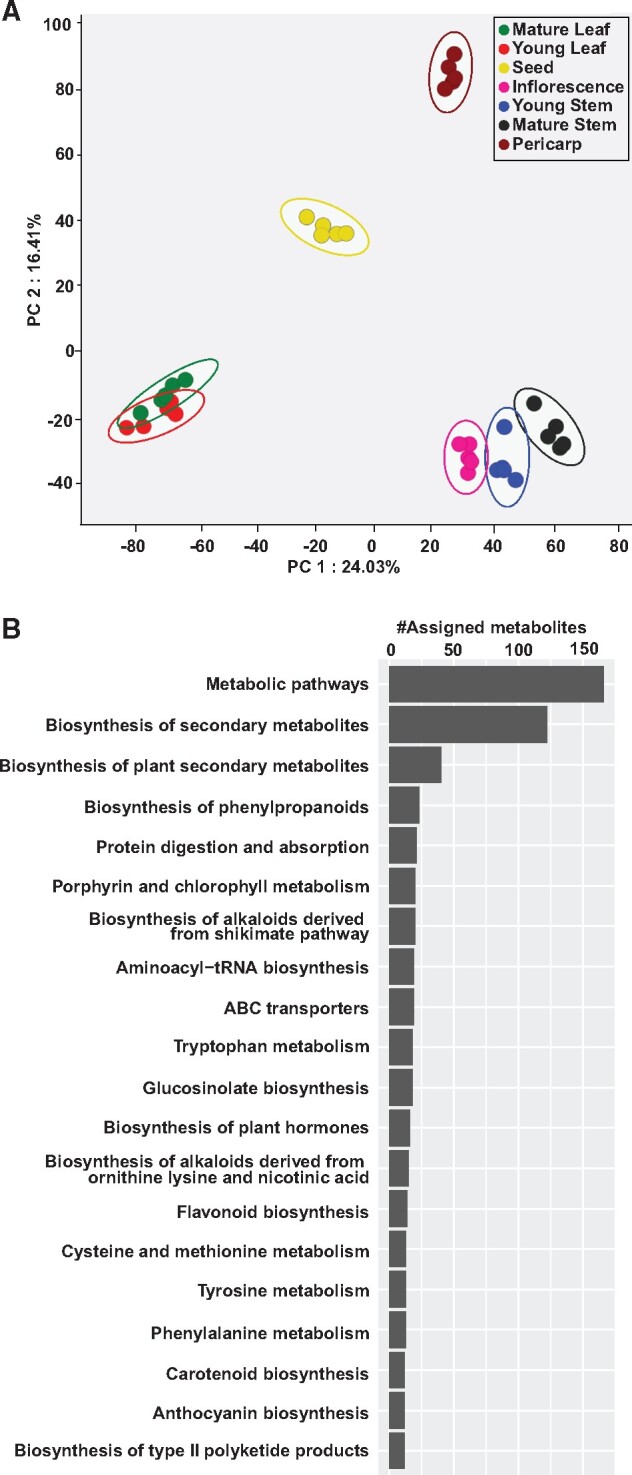
Untargeted metabolome analysis for seven tissues of *Cornus officinalis.* (A) Principal component analysis using metabolome profiling for seven tissues of *C. officinalis.* (B) The top 20 KEGG pathways based on the number of assigned metabolites being detected in this study.

In order to understand the metabo-constitutes of *C. officinalis* across different tissues*,* we extracted metabolites from seven tissues of *C. officinalis* and performed untargeted metabolic profiling (Fig. 1). The acquired raw-data were pre-processed for peak detection and alignment using MS-DIAL,^29^ resulting in a total of 11,017 mass-features in the positive ion mode (Supplementary Table S1). PCA using the normalized intensities for the aligned mass-features showed seven tissues separated as four distinct groups along PC1 and PC2 axes (Fig. 2A). Young leaf and mature leaf were grouped and separated along the PC1 axis with another group constituting young stem, mature stem, and inflorescence. Pericarp and seed tissues were separated along the PC2 axis as distinct tissue groups. The PCA score plot validates the quality of acquired metabolite profiling dataset as we efficiently captured the tissue-wise accumulation of metabolites that contributes to their specific medicinal uses.

In an attempt to gain insights into the overall metabo-constitutes of *C. officinalis*, the acquired mass-features were searched against the KNApSAcK database^30^ with an allowed mass-error window of 10 ppm. In total, we assigned 2,155 mass-features to a corresponding KNApSAcK ID (Supplementary Table S2). Further, to explore the active metabolic pathways in *C. officinalis*, the aligned mass-features were mapped to the KEGG pathway database.^31^ The top three KEGG pathways based on the number of mass-features being assigned included metabolic pathways, biosynthesis of secondary metabolites, and biosynthesis of plant secondary metabolites (Fig. 2B and Supplementary Table S2). The top 20 KEGG pathways assigned to *C. officinalis* metabolome datasets also included several secondary metabolic pathways including biosynthesis of phenylpropanoids, biosynthesis of alkaloids derived from the shikimate pathway, biosynthesis of alkaloids derived from ornithine, lysine, and nicotinic acid, flavonoid biosynthesis, carotenoid biosynthesis, anthocyanin biosynthesis, and biosynthesis of type II polyketide products. Metabolites mapped to the KEGG pathways corresponding to the biosynthesis of alkaloids and flavonoids were highly accumulated in inflorescence, mature stem and young stem (Supplementary Fig. S1A and B and Table S1), while those mapped to the biosynthesis of phenylpropanoids were highly accumulated in pericarp and mature stem (Supplementary Fig. S1C). The majority of the metabolites corresponding to the biosynthesis of terpenoids was highly accumulated in mature leaf and young stem of *C. officinalis* (Supplementary Fig. S1D)*.* Untargeted metabolite profiling using multiple tissues of *C. officinalis* revealed rich chemo-diversity of specialized metabolites accounts for its broad medicinal properties. Against 305 metabolites being previously reported in *C. officinalis,*^3^ the number of mass-features putatively assigned to specialized metabolites in this study suggests the presence of hundreds of metabolites never reported before. While structural determination and compound identification for all of these metabolites seem unfeasible due to technical limitations, nonetheless our data does represent characteristic plant metabolite diversity. The putatively assigned mass-features can serve as candidates for future validation and structural determination, the discovery of which could be prioritized based on its pharmacological importance. Therefore, the metabolome data obtained in this study will be a valuable resource in exploring the metabolic pathways present in *C. officinalis.*

### 3.2. Accumulation of specialized bioactive metabolites in *Cornus officinalis* occurs in a tissue-specific manner

**Figure 3:**
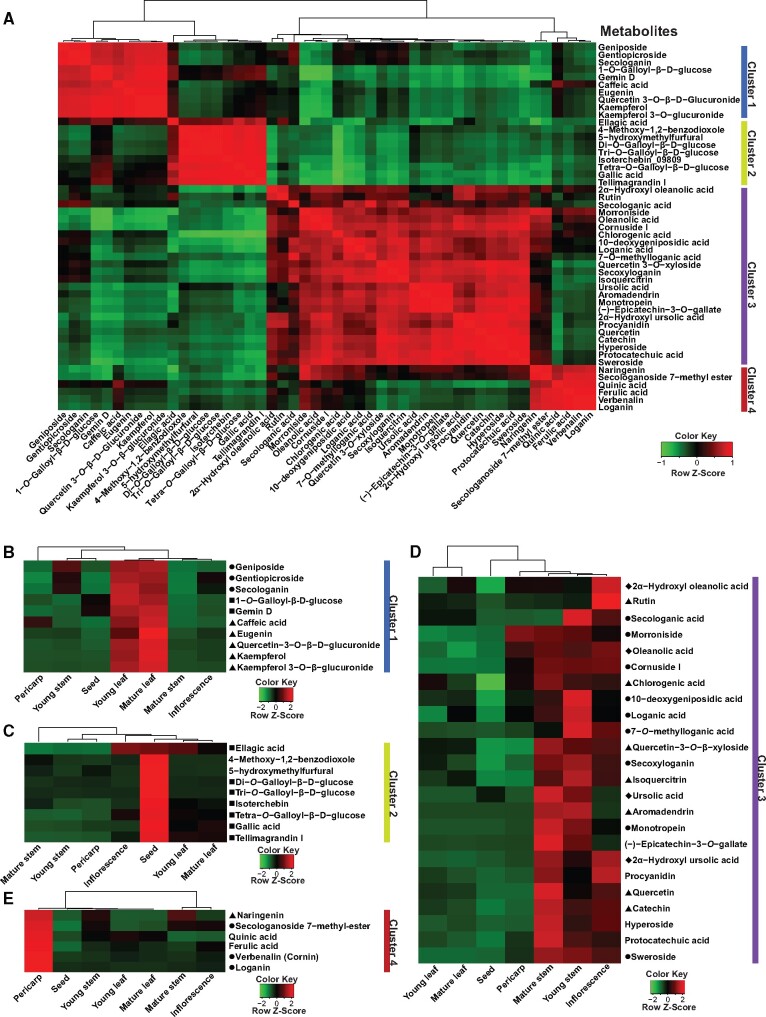
Correlation analysis and accumulation pattern of MS^2^ confirmed metabolites in *Cornus officinalis*. (A) Spearman correlation coefficients were calculated between chemically assigned metabolites using accumulation levels across seven tissues of *C. officinalis*. Correlation scores are plotted as a heatmap with corresponding metabolite names represented along the *X*- and *Y*-axes. Hierarchical clustering based on correlation scores for metabolites formed four distinct groups, named as four metabolite clusters. (B–E) Accumulation of metabolites grouped into four distinct metabolite clusters across seven tissues of *C. officinalis*. Circle, square, diamond, and triangle shapes before the names of chemically assigned metabolites correspond to iridoids, gallotannins, triterpenoids, and phenylpropanoids metabolite families, respectively.

From the mass-features detected in our metabolome dataset, we focused on specialized metabolites reported from *Cornus* genus and identified a total of 49 metabolites through investigation of their MS^2^-based fragmentation pattern summarized in Supplementary Table S3. Correlation based on the accumulation pattern of these 49 metabolites across seven tissues is shown as a heatmap in Fig. 3A. Metabolites were grouped into four clusters, and their accumulation trends were primarily based on the associated chemical families. Metabolites in cluster 1, 50% of which included phenylpropanoids, were highly accumulated in young leaf and mature leaf of *C. officinalis* (Fig. 3B)*.* Cluster 2, predominantly constituted of gallotannins, was highly accumulated in seed except for ellagic acid, which showed the highest accumulation in inflorescence of *C. officinalis* (Fig. 3C). Cluster 3 included all the four triterpenoids and 60% of all the iridoids being identified in this study, with higher accumulation in young stem, mature stem, and inflorescence of *C. officinalis* (Fig. 3D). Morroniside, an iridoid grouped in this cluster, was an exception having the highest accumulation in pericarp. Cluster 4, including two iridoids, namely, loganin and verbenalin together with few other metabolites, was highly accumulated in pericarp of *C. officinalis* (Fig. 3E). Morroniside and loganin, which are the standards described by Chinese pharmacopeia for the assessment of herb quality, were highly accumulated in pericarp and are consistent with the tissue primarily being used for the medicinal purpose.^3^ Correlation of metabolites from the same chemical families have previously been reported in *Arabidopsis*^56^ and tomato,^57^ and our results showed similar metabo-properties in *C. officinalis*.

While our metabolome datasets do reiterate the importance of pericarp of *C. officinalis* for medicinal purpose, the accumulation of important medicinal metabolites including gallotannins and several of the iridoids and triterpenoids having anti-inflammatory, neuroprotective effects, hypoglycaemic, and anti-oxidant activities were also highly accumulated in the other plants’ tissues including stem and leaf. Results obtained from this study, therefore, clearly suggest the importance of different tissues apart from fruits for the extraction of bioactive metabolites.

### 3.3. *De novo* transcriptome assembly established simple sequence repeats database of *Cornus officinalis*

For a comprehensive representation of *C. officinalis* transcriptome, we conducted RNA-sequencing of the same seven tissues that were used for metabolome analysis. The sequencing reads were processed using Trimmomatic programme,^35^ resulting in over 15.1 Gbp clean sequencing reads (Supplementary Table S4). These sequencing reads were subsequently used to derive the *de novo* transcriptome assembly. In order to enhance the contiguity of assembly and maximize the diversity of assembled transcripts, we derived *de novo* transcriptome assembly by concatenating the primary assemblies generated using Trinity,^36^ SOAPdenovo-Trans,^37^ and CLC genomics workbench (https://www.qiagenbioinformatics.com/, 8 May 2020, date last accessed), following which the redundancies were removed using CD-HIT-EST programme.^38^ The final hybrid *de novo* transcriptome assembly included a total of 304,371 unigenes with an average length and N50 as 742 and 1,250 bp, respectively (Table 1). For the quantitative assessment of the *C. officinalis de novo* transcriptome assembly, we performed BUSCO analysis^39^ using 1,614 conserved gene clusters across 60 plant species. BUSCO analysis determined 96% completeness of our assembly, thus demonstrating excellent gene representation of *C. officinalis* transcriptome assembly. Among identified genes, 365 (22.6%) were complete and single-copy genes, 1,184 (73.4%) were completed and duplicated genes, and 34 (2.1%) were fragmented genes (Supplementary Fig. S2A). A high percentage of duplicated genes identified by BUSCO analysis may suggest a potential whole-genome duplication event for *C. officinalis.* The length of the assembled unigenes of *C. officinalis* ranged between 196 and 15,677 bps, with the length of 179,678 unigenes <500 bps and 1,068 unigenes with length over 5,000 bps (Supplementary Fig. S2B).

We scanned the *de novo* transcriptome assembly using MISA software^42^ to identify SSRs in *C. officinalis.* SSRs are short DNA stretches consisting of a 1–6 nucleotide motif repeated, several times in tandem. SSRs undergo rapid mutation resulting in the genetic diversification among closely related species, and hence are considered as a marker for determining genetic variations with close and distant plant species.^58^^,^^59^ In total, 95,533 SSRs were identified, of which di-nucleotide repeat classes formed the largest fraction (47.9%), followed by mono- (40.4%) and tri-nucleotide (10.2%) repeat classes (Table 2). The tetra-, penta-, and hexa-nucleotide repeat classes account for only a small fraction of the total SSRs identified in *C. officinalis*. All the identified SSRs in *C. officinalis* has been listed in Supplementary Table S5. SSRs are widely used to access genetic diversity, for the development of the genetic map, and therefore, represent valuable resources being used for population genetic studies.^60^^,^^61^ The SSRs of *C. officinalis* identified in this study would serve as an important resource for the determination of functional genetic variation in future studies.

### 3.4. Functional characterization and expression analysis using *de novo* transcriptome of *Cornus officinalis*

**Figure 4:**
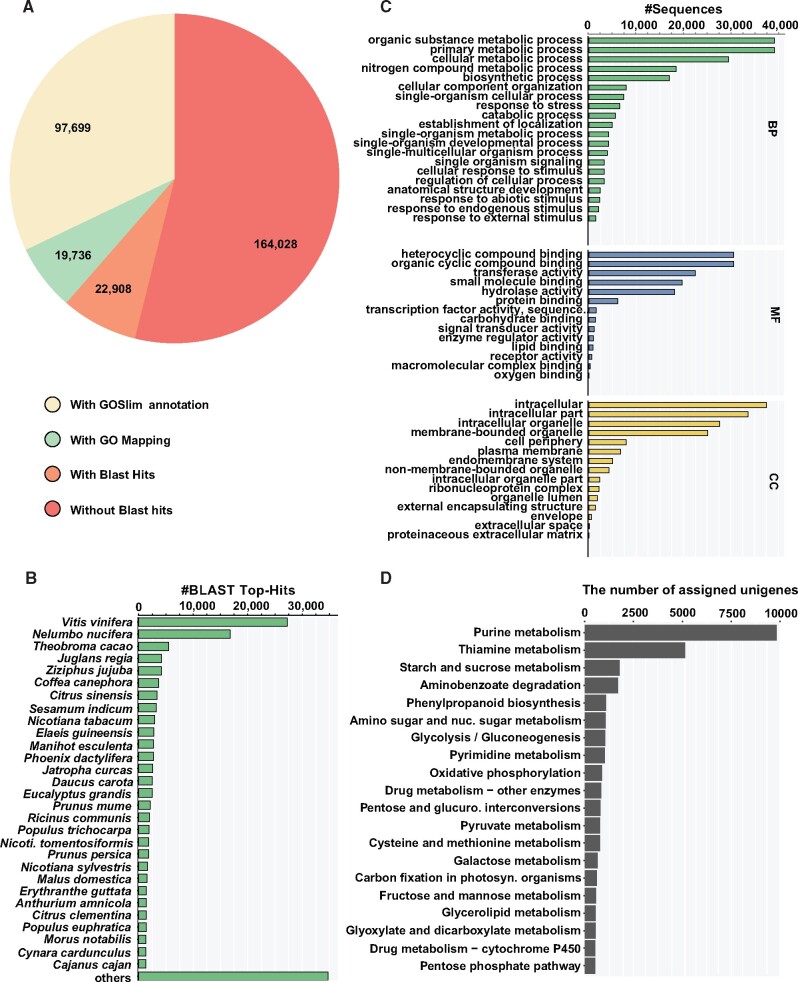
Functional annotation of the *de novo* transcriptome assembly of *Cornus officinalis.* (A) Blastx-based data distribution summary for the annotated transcripts of *C. officinalis.* (B) Blastx top-hit species distribution for the assembled transcripts. (C) Blastx-based GO terms for all the annotated transcripts are summarized in three broad categories; biological processes (BP), molecular function (MF), and cellular components (CC); and (D) top 20 KEGG pathways represented by the annotated transcripts of *C. officinalis* based on the number of assigned transcripts.

The assembled unigenes were subsequently used for annotation to understand their putative function within the biological system. Overall, 140,343 transcripts constituting about 46.1% of the total assembled unigenes were annotated based on their homology search against the NCBI-nr database (http://www.ncbi.nlm.nih.gov, 8 May 2020, date last accessed; formatted on February 2019, Fig. 4A, Supplementary Table S6). Results obtained from Blastx-based annotation showed that the majority of the sequences had significant homology against the sequences present in the NCBI-nr database, with 79,312 unigenes having over 80% sequence-similarity with its corresponding hit (Supplementary Fig. S2C). Top-hit species distribution showed that the assembled unigenes of *C. officinalis* had high sequence-similarity with sequences from *Vitis vinifera, Nelumbo nucifera, Theobromo cacao, Juglans regia,* and *Ziziphus jujube* (Fig. 4B)*.* Despite being an Asterid and from the Cornales order, the first five top-hit species based on Blastx analysis belong to Rosids or early-diverging eudicots, which could very well be due to greater representation of sequenced plant genomes from Rosids clade. The results from Blastx search were used to assign gene ontology (GO) terms, enzyme commission (EC) number, and associated KEGG pathway using BLAST2GO software.^41^ Of the 140,343 annotated unigenes, 117,436 unigenes were assigned with a GO-term under three major GO-categories, namely, cellular component, molecular function, and biological process. The GO-terms having the maximum number of assigned unigenes included intracellular, heterocyclic compound binding, and organic substance metabolic processes under the cellular component, molecular function, and biological process category, respectively (Fig. 4C). KEGG database-based pathway mapping of the assembled unigenes resulted in 33,590 unigenes being assigned to 144 pathways (Supplementary Table S7). Top 20 KEGG pathways based on the number of transcripts being assigned are summarized in Fig. 4D. Annotation and functional characterization of *C. officinalis* transcriptome assembly captured all major biosynthesis pathways and complete coverage of the active biological processes, thus confirming the suitability of this resource to perform down-stream applications, including co-expression and integrative omics analysis.

*De novo* transcriptome assembly was used to perform expression analysis across seven tissues of *C. officinalis.* Among the tissues*,* young stem with 151,729 unigenes and seed with 146,245 unigenes showed the highest number of transcriptionally active unigenes (FPKM > 0). In comparison, mature stem with 70,055 unigenes had the lowest number of transcriptionally active unigenes. The transcriptome profiling revealed that tissues undertaken in this study have relatively distinct ongoing metabolic processes with only 5,969 (1.96%) actively expressed unigenes being shared between seven tissues, while 94,183 (30.94%) unigenes were exclusively expressed in only one of the seven tissues of *C. officinalis* (Supplementary Fig. S3A)*.* These results were consistent with metabolite accumulation analysis, which also showed tissue-specific accumulation of specialized metabolites (Fig. 3)*.* Further, we performed expression-based correlation analysis to understand the global relationship between different tissues of *C. officinalis* at the transcriptome level. Broadly, the correlation analysis revealed two groups with mature stem forming a separate group and the rest of the six tissues forming the second group (Supplementary Fig. S3B). Within the second group, seed and pericarp formed individual groups, while young and mature leaf, as well as young stem and inflorescence, were clustered together. These results indicate that the presence of tissue-specific unigenes associated with each tissue and the overlap of unigenes’ expression can be primarily attributed to their developmental stages and tissue-types. Co-expression analysis showed that the expression for unigenes in *C. officinalis* captured tissue-specific features, and in extension, the biological relevance of key gene expression and associated biological processes. The transcriptome dataset reported in this study, therefore, provides a valuable framework to analyse further the candidate genes participating in the biosynthesis of pharmacologically active metabolites.

### 3.5. Iridoids and triterpenoids showed correlated gene expression and metabolite accumulation in *Cornus officinalis*

Iridoids and triterpenoids are the two most important classes of phytochemicals that attributes to the medicinal properties of *C. officinalis.*^3^^,^^4^ Isopentenyl phosphate and dimethylallyl phosphate, derived from the mevalonate (MVA) pathway and non-mevalonate (MEP) pathway, undergoes cyclization catalyzed either by GPPS or by FPPS to form geranyl diphosphate (GDP) and farnesyl diphosphate (FDP), respectively. GDP and FDP undergo a series of reactions leading to the biosynthesis of iridoids and triterpenoids, respectively. In order to identify the candidate genes associated with the biosynthesis of iridoids and triterpenoids, the transcriptome assembly of *C. officinalis* was screened for transcripts annotated as enzymes involved in these two biosynthetic pathways. In total, we identified 182 and 83 transcripts for triterpenoid and iridoid biosynthetic pathways, respectively (Supplementary Table S8). Previous studies have shown co-expression for genes associated with triterpenoids and iridoids biosynthesis pathways across multiple plant species.^34^^,^^46–52^^,^^62–64^ In order to narrow down genes participating in the triterpenoid/iridoid biosynthetic pathway, we performed correlation cluster analysis of all the transcripts across all the seven tissues.

**Figure 5:**
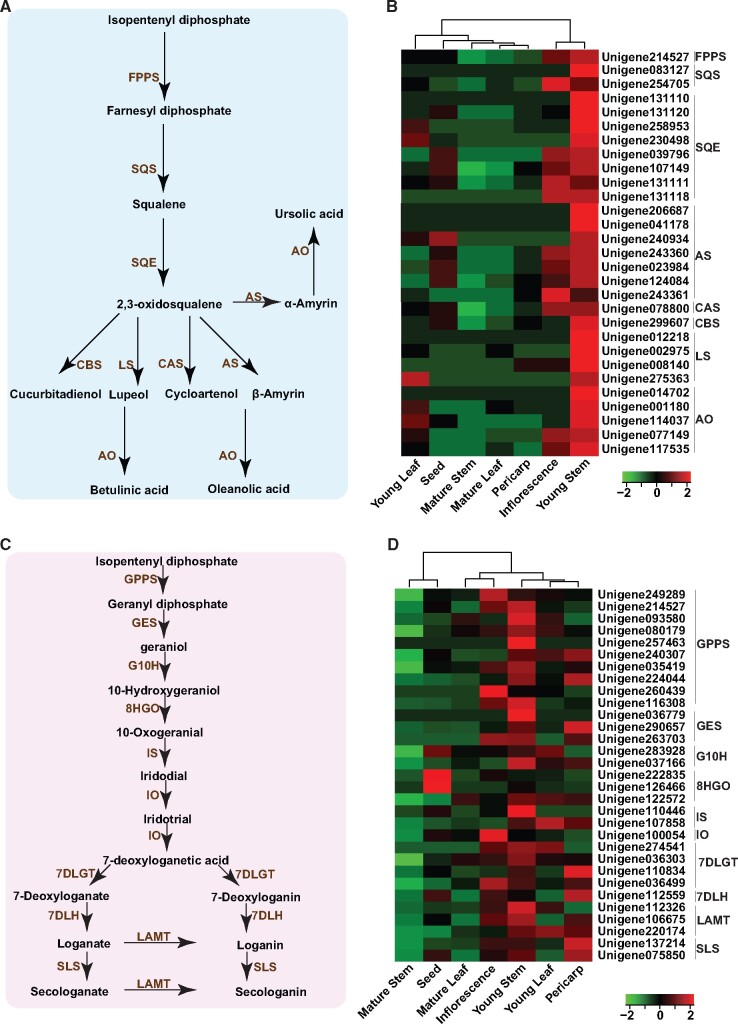
Expression of transcripts assigned to triterpenoids’, and iridoids’ biosynthetic pathways across seven tissues in *Cornus officinalis.* (A) Proposed biosynthetic pathway of triterpenoids; (B) expression pattern of transcripts associated with triterpenoids’ biosynthetic pathway in *C. officinalis*; (C) proposed biosynthetic pathway of iridoids; and (D) expression pattern of transcripts associated with iridoids’ biosynthetic pathway in *C. officinalis*. FPPS, farnesyl diphosphate synthase; SQS, squalene synthase; SQE, squalene epoxidase; CBS, cucurbitadienol synthase; LS, Lupeol synthase; CAS, cycloartenol synthase; AS, amyrin synthase; AO, amyrin oxide; GPPS, geranyl diphosphate synthase; GES, geraniol synthase; G10H, geraniol 10-hydroxylase; 8HGO, 8-hydroxygeraniol oxidoreductase; IS, iridoid synthase; IO, iridoid oxidase; 7DLGT, 7-deoxyloganetic acid glucosyltransferase; 7DLH, 7-deoxyloganic acid hydroxylase; LAMT, loganic acid *O*-methyltransferase; SLS, secologanin synthase.

Correlation analysis based on the expression of transcripts annotated as enzymes associated with the triterpenoid biosynthetic pathway followed by hierarchical clustering revealed a highly co-expressed gene cluster including 29 transcripts, representing eight known enzymes from the triterpenoid biosynthetic pathway (Fig. 5A and Supplementary Fig. S4). Therefore, we selected these 29 transcripts as the candidate genes associated with the biosynthesis of triterpenoids in *C. officinalis*. The expression of these candidate transcripts was highest in the young stem, followed by inflorescence, while low expression was detected for seed, leaf, and pericarp (Fig. 5B). The expression trends of the candidate genes were consistent with the accumulation of all the four triterpenoids, namely, ursolic acid, oleanolic acid, 2α-Hydroxy ursolic acid, and 2α-Hydroxy oleanolic acid (Supplementary Fig. S5). Therefore, we assigned these 29 transcripts as the candidate genes associated with the biosynthesis of triterpenoids in *C. officinalis.*

Similar, correlation analysis of the transcripts annotated as homologs from iridoid biosynthetic pathway followed by hierarchical clustering showed the presence of a highly co-expressed gene cluster including 31 transcripts, representing all 10 known enzymes of iridoid biosynthetic pathway (Fig. 5C and Supplementary Fig. S6). These 31 genes were selected as the potential candidate genes associated with the biosynthesis of iridoids in *C. officinalis*. Expression analysis of these candidate transcripts showed the highest expression in the young stem, followed by inflorescence and pericarp (Fig. 5D). Similar to the expression trend of candidate transcripts, the metabolome analysis also showed a consistent accumulation trend for the majority of iridoids identified in this study (Supplementary Fig. S7). The accumulation of loganin and morroniside, the two principal pharmaceutical compounds of *C. officinalis,* was highest in pericarp and is in accordance with the tissue being primarily used for medicinal purposes. While our study indicates the significance of *C. officinalis* fruits for medicinal purposes, it also suggests the relevance of young stem and inflorescence for use in traditional medicine formulations or for deriving important medicinal compounds from *C. officinalis*.

### 3.6. Transcripts expression and metabolome analysis suggest different sites for the biosynthesis and accumulation of gallotannins in *Cornus officinalis*

**Figure 6:**
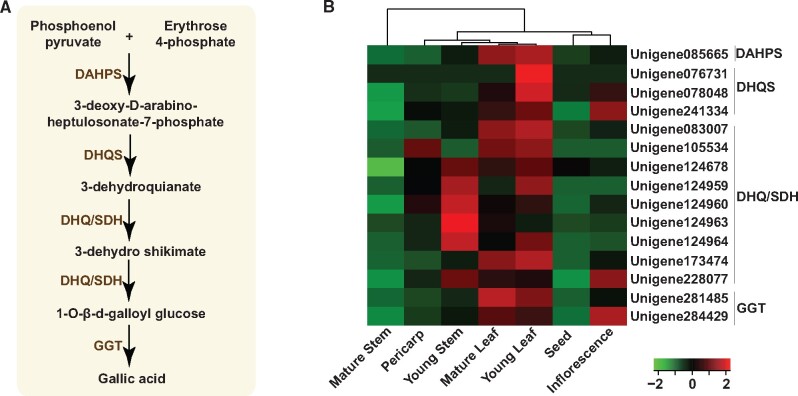
Expression of transcripts assigned to gallotannins’ biosynthetic pathway across seven tissues in *Cornus officinalis*. (A) Proposed biosynthetic pathway of gallotannins*;* (B) Expression pattern of transcripts associated with gallotannins’ biosynthetic pathway in *C. officinalis*. DAHPS, 3-Deoxy-D-arabinoheptulosonate 7-phosphate synthase; DHQS, 3-dehydroquinate synthase; DHQ/SDH, 3-dehydroquinate dehydratase/shikimate dehydrogenase; and GGT, gallate glucosyltransferase.

Gallotannins, which contains at least one moiety each of galloyl and sugar/cyclitol, are essential constituents of the metabolite pool of *C. officinalis.* They are known to impart anti-oxidative, neuroprotective, and hepatoprotective properties to *C. officinalis.*^16^^,^^17^^,^^65^ In order to identify transcripts associated with biosynthesis of gallotannins in *C. officinalis,* the transcriptome assembly was searched for the transcripts annotated as enzymes known to be involved in the biosynthesis of gallotannins (Fig. 6A). In total, we identified 37 transcripts, correlation analysis of which revealed a highly co-expressed gene cluster of 15 transcripts, representing five known enzymes of gallotannins biosynthetic pathway (Supplementary Fig. S8 and Table S9). Therefore, these 15 transcripts were selected as candidate genes associated with the biosynthesis of gallotannins in *C. officinalis* (Fig. 6B). While the majority of the candidate transcripts was highly expressed in young leaf, followed by mature leaf and inflorescence, the majority of gallotannins identified in the metabolome dataset showed the highest accumulation in the seed, which is in accordance with the findings, reported earlier^66^ (Supplementary Fig. S9). Few of the gallotannins, including gemin D, tellimagrandin I, and 1-*O*-galloyl-β-D-glucose, were significantly accumulated in young leaf and mature leaf. These results suggest that the early precursors in the biosynthesis of gallotannins are probably synthesized in the leaf tissue, which is then transported to the seeds where further synthesis and modifications occur. Generally, tannins are known to accumulate in the seeds, while the biosynthesis pathway is localized to leaves and other tissues.

### 3.7. Network-based integrative omics analysis reveals seven cytochrome P450s involved in the metabolism of iridoids

**Figure 7:**
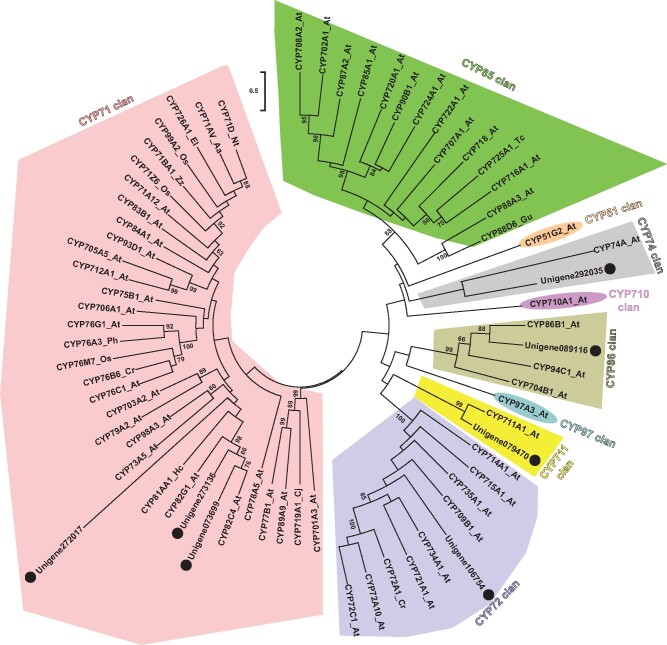
Phylogenetic analysis of the candidate transcripts annotated as cytochrome P450s in *Cornus officinalis* together with CYPs representing major-classes of plant CYPs. Seven transcripts (represented with a black circle) were highly correlated with at least 50% of assigned iridoid biosynthetic genes as well as with at least one iridoid of a total 15 being identified in this study. Nucleotide sequences were translated to their corresponding protein sequences and aligned using MUSCLE programme. The phylogenetic tree was inferred using the Maximum Likelihood method with bootstrap values obtained after applying 1,000 replications using the MEGA X programme. Bootstrap values above 60% are shown here. The tree is drawn to scale, with branch lengths measured in the number of substitutions per site. Evolutionary analyses were conducted in MEGA X. At, *Arabidopsis thaliana*; Os, *Oryza sativa*; Cj, *Coptis japonica*; El, *Euphorbia lagascae*; Tc, *Taxus cuspidata*; Nt, *Nicotiana tabacum*; Aa, *Artemisia annua*; Zz, *Zingiber zerumbet*; Cr*, Catharanthus roseus*; Gu, *Glycyrrhiza uralensis*; Hc, *Hypericum calycinum*; Ph, *Petunia* x *hybrida.*

Cytochrome P450s play a vital role in the diversification of metabolite pool via catalyzing modification reaction, including oxidation, hydroxylation, alkylation, and esterification of the core skeleton molecule.^67^^,^^68^ While several previous studies and our results have shown the presence of a diverse range of iridoids in *C. officinalis,*^3^^,^^4^^,^^69–71^ there exists limited information on the CYPs that may participate in its biosynthesis and diversification. Therefore*,* we adopted a metabolome guided approach to assign candidate CYPs to the iridoid biosynthetic pathway. The *de novo* transcriptome assembly was screened to identify transcripts annotated as cytochrome P450s, resulting in a total of 822 transcripts. Among these, 262 CYPs with a length of at least 500 bps and FPKM value of 5 in at least one of the seven tissues were selected for subsequent analysis (Supplementary Table S10). These selected CYPs were used to perform Spearman’s data matrix-based correlation together with the identified iridoids in our metabolome dataset, which showed 45 transcripts to be highly correlated with at least one of the 15 iridoids with *R*^2^ > 0.85 (Supplementary Fig. S10 and Table S11). Among transcripts, seven were highly correlated with at least 50% of the assigned biosynthetic enzymes, and therefore represent strong candidates with a putative function in the iridoids biosynthesis and diversification in *C. officinalis.* Subsequently, phylogenetic analysis of these seven transcripts was performed together with 61 CYPs representing all the known clans of cytochrome P450s across different plant species. Phylogenetic analysis revealed that three of the seven transcripts were grouped in CYP71-clan (A-type), while remaining of them were grouped in CYP72-, CYP74-, CYP711-, and CYP86-clan, respectively (Fig. 7). CYP71-clan represents the largest clan of CYP constituting over 50% of the plant CYPs, members of which are known to have a diverse range of functions, particularly in plant’s specialized metabolism. CYP71-clan is exclusive to the land plants and is reported to have undergone several successive gene duplication events resulting in the evolution of species-specific novel genes participating in plant’s secondary metabolism.^67^^,^^68^ Within the transcripts grouped in the CYP71-clan, two of them were phylogenetically close to CYP82C4 and CYP82G1 of Arabidopsis, and CYP81AA1 of *Hypericum calycinum.* CYP82C4 and CYP82G1 catalyze the hydroxylation reaction of 8-methoxypsoralen, and cleavage reaction of (*E, E*)-geranyllinalool to yield homoterpene volatiles, respectively.^72^ CYP81AA1 is known to participate in the biosynthesis of plant xanthones.^73^ Previous studies have reported diverse functions, including hydroxylation and demethylation with a role in alkaloid biosynthesis, for members of the CYP82 family.^74–76^ The wide range of activity and substrate-specificity for members of CYP82 family within CYP71-clan may suggest that three transcripts of *C. officinalis* grouped herein may have a potential role in the biosynthesis of iridoids in *C. officinalis*. Among the transcripts grouped within non-A type CYPs, Unigene106754 was clustered in the CYP72-clan. CYP72A1 from *Catharanthus roseus* is known to participate in secologanin biosynthesis^77^ and, therefore, Unigene106754 represents a prominent candidate that may participate in the biosynthesis of iridoids in *C. officinalis.* Phylogenetic analysis of the transcripts annotated as cytochrome P450s, as narrowed down by the metabolome guided approach, facilitated the identification of three CYPs representing potential candidates participating in the biosynthesis of iridoids in *C. officinalis.* Further functional characterization of the identified CYPs will help in ascertaining their role in the iridoids’ metabolism in *C. officinalis.*

## 4. Conclusion

In this study, we investigated the biosynthesis of pharmacologically important specialized metabolites in *C. officinalis* through RNA-sequencing and untargeted metabolome analyses. We identified 1,215 metabolites via mapping the mass-features on to the KEGG pathway database and reported the accumulation pattern of MS^2^ confirmed 49 metabolites across seven tissues of *C. officinalis*. Our results showed that not only the fruit, the tissue used for all major traditional medicine purposes, but also other tissues such as young leaves and stems are an equally potent source of medicinal compounds. We used multi-omics analysis to identify candidate genes involved in the biosynthetic pathway of iridoids, triterpenoids, and gallotannins, the dominant group of bioactive metabolites in *C. officinalis*. Correlation-based integration of the transcriptome and metabolome dataset and network-based analysis helped in the identification of 45 cytochrome P450s showing a high correlation with iridoids. Further analyses of those highly correlated CYPs strongly suggested the putative involvement of three CYPs participating in the iridoid metabolisms in *C. officinalis*. Besides, the transcriptome and metabolome resources being established in this study will lay the foundation for screening novel metabolites present in *C. officinalis* and future studies on their biosynthesis through functional characterization of associated transcripts discovered in this study.

### 4.1. Data availability

The raw sequence reads for all seven tissues of *C. officinalis*, their expression value and the *de novo* transcriptome assembly used in this study have been deposited in NCBI’s Gene Expression Omnibus (GEO) and are available at GEO series accession number GSE144198. Plant material used in this study and raw metabolome dataset is available from the corresponding authors on request.

## Supplementary data

Supplementary data are available at *DNARES* online.

## Supplementary Material

dsaa009_Supplementary_Data
