# Supplementary material for: Multiomics-based characterization of specialized metabolites biosynthesis in Cornus Officinalis
Source: DNA Res. 2020 May 19;27(2):dsaa009. doi: 10.1093/dnares/dsaa009 (PMC7320821; doi:10.1093/dnares/dsaa009)
Supplement: dsaa009_Supplementary_Data [file dsaa009_supplementary_data.zip › Supplementary_information_legends_revised.doc]

**Supplementary information legends**

**Supplementary Figure 1: KEGG pathway mapping of candidate metabolites.** The chemical identities to the mass-features were assigned using a mass-error window of 10 ppm against the KEGG database, and candidate metabolites were grouped as (A) Biosynthesis of alkaloids; (B) Biosynthesis of flavonoids; (C) Biosynthesis of phenylpropanoids; and (D) Biosynthesis of terpenoids. The relative peak area for a given metabolite in tissue was calculated as a percentage of its cumulative peak area across all seven tissues of *Cornus officinalis* and shown along the X-axis.

**Supplementary Figure 2: Functional characterization of the assembled *de novo* transcriptome assembly of *Cornus officinalis.*** (A) BUSCO analysis of the *de novo* transcriptome assembly of *C. officinalis*; (B) Length distribution of the assembled transcripts; (C) Sequence-similarity distribution of the assembled transcripts using the top blastx hits.

**Supplementary Figure 3: Transcriptome analysis across seven tissues of *Cornus officinalis.*** (A) Venn-diagram for the number of active transcripts shared and unique across each tissue of *C. officinalis;* (B) Transcripts expression across each tissue was calculated in terms of FPKM values and were then used for Pearson-based correlation analysis.

**Supplementary Figure 4: Correlation plot for unigenes annotated as enzymes associated with triterpenoids’ biosynthetic pathway.** Correlation analysis was performed using transcriptome expression data (FPKM) across all seven tissues. Hierarchical clustering identified complete triterpenoids’ biosynthetic pathway within a highly co-expressed gene-cluster (shown with a purple-colored box).

**Supplementary Figure 5: Accumulation of triterpenoids identified in this study across seven tissues of *Cornus officinalis*.** The median value of intensities (as total peak area) was used to draw the bar diagram while the standard deviation was calculated based on variance across five biological replicates.

**Supplementary Figure 6: Correlation plot for unigenes annotated as enzymes associated with iridoids’ biosynthetic pathway.** Correlation analysis was performed using transcriptome expression data (FPKM) across all seven tissues. Hierarchical clustering identified complete iridoids’ biosynthetic pathway within a highly co-expressed gene-cluster (shown with a purple-colored box).

**Supplementary Figure 7: Accumulation of iridoids identified in this study across seven tissues of *Cornus officinalis*.** The median value of intensities (as total peak area) was used to draw the bar diagram while the standard deviation was calculated based on variance across five biological replicates.

**Supplementary Figure 8: Correlation plot for unigenes annotated as enzymes associated with gallotannins’ biosynthetic pathway.** Correlation analysis was performed using transcriptome expression data (FPKM) across all seven tissues. Hierarchical clustering identified complete gallotannins’ biosynthetic pathway within a highly co-expressed gene-cluster (shown with a purple-colored box).

**Supplementary Figure 9: Accumulation of gallotannins identified in this study across seven tissues of *Cornus officinalis*.** The median value of intensities (as total peak area) was used to draw the bar diagram while the standard deviation was calculated based on variance across five biological replicates.

**Supplementary Figure 10: Network diagram based on correlation values between iridoids (green) and transcripts annotated as cytochrome P450s (blue).** The networks were visualized with the Cytoscape software (version 3.7.2). The CYPs, which have a high correlation coefficient with iridoids and with at least 50% of the assigned iridoids biosynthetic genes, are highlighted in brown. The color of the connection lines is metabolite-specific. All the unigenes shown in the figure shows a high correlation coefficient with at least one of the iridoids (R^2^>0.85). While the metabolites are connected with each other if they have R^2^>0.8, they are connected with transcripts if R^2^>0.6.

**Supplementary Table 1: Comprehensive list of mass-features identified in this study and their intensity level (as total peak area) across seven tissues of *Cornus officinalis,* with five biological replicates for each tissue**.

**Supplementary Table 2: KEGG database, and KNApSAcK database mapping of the identified mass-features in *Cornus officinalis*.**

**Supplementary Table 3: Fragmentation pattern of MS^2^ confirmed 49 metabolites.** Columns in the table include metabolite ID, parent ion (*m/z* value), retention time, product ions, and total peak area across seven tissues of *Cornus officinalis*, five biological replicates for each tissue.

**Supplementary Table 4: Summary of Trimmomatic program based pre-processing of raw reads for all seven tissues of *Cornus officinalis.***

**Supplementary Table 5: List of identified SSRs in *Cornus officinalis de novo* transcriptome assembly.**

**Supplementary Table 6: Blastx based annotation for the *de novo* transcriptome assembly of *Cornus officinalis.***

**Supplementary Table 7: A comprehensive list of KEGG pathways represented in the *Cornus officinalis* *de novo* transcriptome assembly.**

**Supplementary Table 8: List of transcripts, annotated based on sequence similarity as enzymes known to be involved in the biosynthesis of triterpenoids and iridoids, and its expression values (FPKM) across seven tissues of *Cornus officinalis*.**

**Supplementary Table 9: List of transcripts, annotated based on sequence similarity as enzymes known to be involved in the biosynthesis of gallotannins, and its expression values (FPKM) across seven tissues of *Cornus officinalis*.**

**Supplementary Table 10: Transcripts annotated as cytochrome P450s with length over 500 bp and FPKM value at least 5 in one of the seven tissues of *Cornus officinalis*.**

**Supplementary Table 11: Expression (FPKM) values of cytochrome P450s annotated unigenes having high-correlation value (R^2^>0.85) with at least one of the iridoids identified in this study.**
