# Supplementary material for: Multiomics-based characterization of specialized metabolites biosynthesis in Cornus Officinalis
Source: DNA Res. 2020 May 19;27(2):dsaa009. doi: 10.1093/dnares/dsaa009 (PMC7320821; doi:10.1093/dnares/dsaa009)
Supplement: dsaa009_Supplementary_Data [file dsaa009_supplementary_data.zip › Supplementary_Table 4 Summary of Trimmomatic output for Cornus officinalis_revised.doc]

**Supplementary Table 4: Summary of Trimmomatic program based pre-processing of raw reads for all seven tissues of *Cornus officinalis.***

|  | **Young Leaf** | **Mature Leaf** | **Inflorescence** | **Pericarp** | **Seed** | **Young Stem** | **Mature Stem** |
| --- | --- | --- | --- | --- | --- | --- | --- |
| **Input Read Pairs** | 11,812,990 | 12,186,356 | 9,283,919 | 8,855,191 | 12,664,215 | 13,486,487 | 7,678,545 |
| **Both Surviving** | 10,757,000 (91.06%) | 11,034,756 (90.55%) | 8,393,448 (90.41%) | 8,068,680 (91.12%) | 11,539,190 (91.12%) | 13,100,709 (97.14%) | 6,896,946 (89.82%) |
| **Forward Only Surviving** | 725,891 (6.14%) | 804,846  (6.60%) | 487,398 (5.25%) | 535,368 (6.05%) | 769,522 (6.08%) | 0 (0.00%) | 440,438 (5.74%) |
| **Reverse Only Surviving** | 212,277 (1.80%) | 218,815 (1.80%) | 334,197 (3.60%) | 160,782 (1.82%) | 225,928 (1.78%) | 0 (0.00%) | 276,696 (3.60%) |
| **Dropped** | 117,822 (1.00%) | 127,939 (1.05%) | 68,876 (0.74%) | 90,361 (1.02%) | 129,575 (1.02%) | 385,778 (2.86%) | 64,465 (0.84%) |
